# News and Events of the Week

**Published:** 1909-10-30

**Authors:** 


### News and Events of the Week.

The annual meeting of the Royal College of Surgeons will be held on Thursday, November 18.

Mr. Stansfield Collier, F.R.C.S., has been elected Honorary Consulting Surgeon to the Cranleigh Cottage Hospital.

A fresh case of Asiatic cholera is reported from Germany, this time at Tilsit. The victim is a boatwoman from Tapiau.

The Croydon Council has authorised an expenditure of £250 for the provision of furniture, utensils, and service for providing food for underfed school children. The cost of the food is again to be paid from a voluntary fund, for the collection of which a ward committee has been formed.

Owing to unforeseen material difficulties, and in order to give its due importance to the exhibition attached to the third International Congress of School Hygiene, the French Organising Committee has decided to postpone the date of the Congress until the first week of August (2nd to 7th) 1910.

Professor J. A. Lindsay will deliver the Bradshaw Lecture at the Royal College of Physicians on November 2, taking for his subject "Darwinism and Medicine," and Professor Sir T. Clifford Allbutt will deliver the Fitz-Patrick Lecture on "Greek Medicine in Rome" on November 4 and 9.

A writer in the *Veterinary Surgeon* states that he has for several years painlessly destroyed animals by the injection of fulminating doses of Sol. strychnia. For horse 10 grs., for dog or cat 2 grs., injected between two ribs into the pleural cavity, or injected intravenously. The method is stated to be humane, quick, and cleanly.

The 20th annual report of the Asylums Committee of the London County Council shows that the number of lunatics on January 1 last, for whose accommodation the Council was primarily responsible, was 19,716, of whom 8,518 were males and 11,198 females. Of the total number, 18,768 (8,115 males and 10,653 females) were parish patients in London County asylums. The report, which is a bulky volume of 300 pages, contains a vast amount of detailed information respecting the management and control of the asylums, and the statistics are particularly full and adequate.

The Autumn issue of *Night and Day*, the quarterly official organ of Dr. Barnardo's Homes, is full of interesting matter, not only for the many supporters of the Institutions, but for all who keep an eye on the progress of one of the most remarkable philanthropic agencies of our day. The Homes have 8,500 boys and girls under their daily charge, and they have rescued in the course of their forty-three years of existence 67,634 children. The report mentions that two of the projects which the management has at heart are the establishment of a Boys' Garden City on an estate at Woodford Bridge, and the erection of the long-projected Hospital at the Girls' Village Home. A copy of this issue will be sent gratis and post free to any applicant who writes to the Honorary Secretary of the Homes at the well-known address, 18 to 26 Stepney Causeway, London, E.

The Amersham Guardians have refused a request of a medical officer (Dr. Wynne) for an increase in salary owing to the higher cost of the drugs in consequence of the Budget.

Dr. Willoughby Marston Garsia, of Woodford, Clarence Park, Weston-super-Mare, who died on September 20, left estate valued at £24,575 gross, with net personalty £24,404.

The Oakland College of Medicine, California, has founded a chair in Tropical Medicine. Dr. Creighton Wellman has been appointed to the professorship of the new department.

Dr. John Hodgson Waterhouse, of Avenue Victoria, Scarborough, formerly house surgeon to the Liverpool Northern Hospital, left estate valued at £43,997 gross, with net personalty £43,927.

The medical officer at Aston reported a case in which a post-mortem on an old woman showed a globular mass about the size of a cricket ball in the stomach. The deceased had worked in the wire trade and had been in the habit of eating particles of copper wire, which had formed into a mass.

Dr. G. H. Savage delivered the Harveian Oration at the Royal College of Physicians on Monday, the 18th October, Sir William Church, Bart., the President, occupying the chair. At the conclusion of the oration the President presented the Moxon medal to Sir William Gowers, the Baly medal to Professor Emil Fischer, of Berlin, who received the medal by deputy, and the Weber-Parkes prizes to Dr. Wilkinson and Dr. Inman.

The Council of the Royal College of Surgeons has made arrangements for six demonstrations upon specimens contained in the museum to be delivered at the College by Professors Arthur Keith and S. G. Shattock, the Conservator and Pathological Curator respectively. The demonstrations to take place on each Friday and Monday afternoon, began yesterday, and will be open to students, medical practitioners, and others interested in physiology and surgical pathology.

Sir Shirley Murphy, in a report to the London County Council, thinks that the recent scarlet fever epidemic in London and the Thames Valley was due to infection derived from the morbid condition of a newly calved cow on a particular farm. Four hundred people were affected by the outbreak. Careful investigation has shown that, although scarlet fever did occur in the family of a milker, it broke out at a date subsequent to the infection of the milk consumers, and was probably also due to the consumption of the milk.

### APENTA SPRINGS.

Members of the recent International Medical Congress took an opportunity during their stay at Budapest of visiting the "Apenta" springs where the process of raising the bitter waters was shown, together with the ingenious machinery employed. The visitors were impressed by the scrupulous cleanliness that prevails throughout and also by the fact that the water does not come into contact with the hands of the workers. Only healthy persons are employed on the premises. During three or four days parties consisting of several hundred members of the Congress went to see the wells.

An International Congress of Otology will be held at Boston in August 1912.

An epidemic of infantile paralysis has been so severe in St. Paul, U.S.A., that the schools have remained closed in consequence.

The annual dinner of the London School of Tropical Medicine took place last Tuesday evening at the Savoy Hotel. The School has lately received an endowment of £1,200 from Lord Sheffield, which this year is to be applied to the study of diphtheria in Fiji.

The Town Council of Glasgow has discussed a recommendation by its Health Committee that consumption be made a compulsory notifiable disease in the city for a period of three years. Dr. Chalmers, the medical officer of health, estimates that the adoption of the scheme proposed will involve an annual expenditure of about £1,380.

The Commissioners of the District of Columbia, U.S.A., have arranged for the prohibition or regulation of the sale or use of dangerous hair-washes. The attention of the Commissioners was called to the subject by a report from the health officer, based on cases in the district, in which women were poisoned through the use by hair dressers of a toxic substance in giving a dry shampoo.

The National Food Reform Association, of 178 St. Stephen's House, Westminster, has just issued its first annual report, which, besides containing a record of work done, includes unpublished letters of much interest from Mr. Seebohm Rowntree and others, emphasising the urgency of the question of diet reform in its relation to the physical condition of the people of Great Britain. Copies, price 3d., may be obtained from the Secretary.

At a meeting of the Faculty of Medicine of the Liverpool University, held on October 22, the Dean announced the entries for the Winter Session as follows: Medical School.—New entries for Degree Courses, 23; new entries for Diploma Courses, 8, total 31; total number of undergraduates registered, 132; new entries for the Diploma of Tropical Medicine, 19; new entries for the Diploma of Public Health, 13. Dental School.—New entries for degrees and diplomas, 14; total number of dental students registered, 42.

The British Fire Prevention Committee has conducted a further series of fire tests with flannelette at the Regent's Park testing station, the number of specimens that have now been under review numbering over 400. The reason for these tests is the great number of fatalities arising from flannelette catching fire. Ordinary flannelette, fine flannelette, non-inflammable flannelette, union and wool materials were under test in lengths of one or two yards, and in the form of garments on dummies representing children. The materials had been washed at three different laundries five, ten, and twenty times respectively. The afternoon's operations went to show that there is certainly a non-flam treatment available of practical value, by which flannelette can be made effectively flame-proof. The object of the tests was to endeavour to obtain precise data as to the fire-resistance of treated flannelette after many washings, as also to devise some easy method of rapidly proving the precise resistance of flannelette sold as "non-inflammable," the importance of the question of preventing the sale of bogus "fire-resisting" materials being considerable. The tests were conducted under the committee's usual procedure. A report on the subject illustrated by diagrams, will be shortly issued by the committee.

The Duchess of Albany last Saturday attended a meeting at St. Peter's Hall, Brockley, in support of the work of the Deptford branch of the League of Mercy. Her Royal Highness subsequently patronised a sale of work in support of the League, and received a large number of vice-presidents and members who have been added to the roll during the past year.

Professor Osler, at the London School of Tropical Medicine, Connaught Road, Albert Dock, last Tuesday opened the winter session by an address on "The Nation and the Tropics." He said that in order to secure health in the Tropics two things were essential: organised centres from which the work of research might proceed, and the unification of these centres under a central tropical institute. He instanced the Sleeping Sickness Bureau under the auspices of the Royal Society as an existing model of what should be done, and said that tropical sanitation would loom larger and larger in the future.

### LEPROSY IN CYPRUS.

According to a report from the Colonial Office, presented to the Second International Scientific Conference now sitting at Bergen, there is no special grouping of leprosy cases in Cyprus, cases being found with equal frequency on the littoral and inland. It is currently believed by the Cypriots that the use of olive oil in quantity as a food results in leprosy, and in support of this it is stated that the chief oil-producing villages of Cyprus contribute more lepers than the other villages in proportion to population. Moslems, whose religion forbids the use of pork, suffer from leprosy in practically the same proportion to population as do the pork-eating Orthodox Christians. In Gibraltar Dr. Turner met with only three cases of leprosy in twenty-seven years. In Malta all the lepers belong to the Maltese lower classes. Thirty cases admitted the existence of leprosy in their family history; forty-three patients used salt fish as one of their principal articles of food, and four never used it. In the Gambia there is at least one leper in every town of any size, and the natives make hardly any attempt to isolate those suffering from the disease, although many understand that it is contagious. A leper, as long as he is able, pursues his ordinary business, and lives with his family and wives. Although the people near the sea probably eat more fish than elsewhere, there is a large trade all up the river in dried fish, which is one of the favourite relishes for the natives' ordinary dishes of rice or meal. Dr. Macfarlane, in his report on the prevalence of leprosy in North Basutoland, states that the disease has nothing to do with salt-fish-eating habits of the Basuto, that there are few or no facilities for the supply of that commodity, that their ancestral habits are against fish-eating, and that the disease spreads from one person to another and from one place to another by contagion. In one instance, a leper Zulu doctor infected a family, and, subsequently, many people living in a village at Sebetoane. Five survive now, and about a dozen have died within the last ten years. As regards contagion, Dr. Macfarlane cites some cases which seem to him to be conclusive on the point. The medical officer of the Bechuanaland Protectorate, Dr. D. M. MacRae, reports that his experience, extending over five and a half years, includes only two cases of leprosy. He does not at all favour the fish-eating theory. In fact, the greatest diversity of evidence and opinion seems to prevail as to the ætiology of the disease. In Southern Rhodesia, East Africa, Nyasaland, Uganda, the Bahamas, British Guiana, Jamaica, the Leeward Islands, Trinidad, Ceylon, Hong Kong, Mauritius, the Straits Settlements, and Fiji, the disease has made its appearance under the most varying conditions as to situation, soil, and food.

